# Retinitis Pigmentosa and Bilateral Idiopathic Demyelinating Optic Neuritis in a 6-Year-Old Boy with* OFD1* Gene Mutation

**DOI:** 10.1155/2017/5310924

**Published:** 2017-01-16

**Authors:** Xun Wang, Cong Zheng, Wen Liu, Hui Yang

**Affiliations:** Department of Fundus Diseases, Zhongshan Ophthalmic Center, Sun Yat-Sen University, 54 S. Xianlie Road, Guangzhou 510060, China

## Abstract

To identify the cause of a sudden binocular vision decrease in patients with retinitis pigmentosa and bilateral idiopathic demyelinating optic neuritis is difficult, but early diagnosis and treatment significantly improve the prognosis. Here, we report a 6-year-old boy with a progressive binocular vision decrease in 38 days. The patient had a history of night blindness, a mottled retina without pigmentation, extinguished electroretinographic response, tritanopia, and an absent ellipsoid zone outside the macula fovea by optical coherence tomography in both eyes. His condition was diagnosed as retinitis pigmentosa (RP) with idiopathic demyelinating optic neuritis (IDON). After corticosteroid therapy, visual acuity recovered to OD: 0.5 and OS: 0.4. Genetic analysis revealed a G985S variant in the oral-facial-digital syndrome 1 gene. Ophthalmologists should pay attention to the existence of other complications in patients with RP who suffer a sudden decrease in vision. A gene survey can help clarify this diagnosis. To our knowledge, this is the first report of a patient with RP and ON, as well as genetic testing results. Nevertheless, the pathogenicity of the variant needs further confirmation.

## 1. Introduction

Retinitis pigmentosa (RP) is an inherited progressive retinal degenerative disease with an incidence of one in every 3,000 to 5,000 individuals [1]. Idiopathic demyelinating optic neuritis (IDON) is a demyelinating autoimmune disease of optic nerve, characterized by acute vision loss. There has been a report of monocular IDON in patients with RP only once before [2]. To the best of our knowledge, there have never been any reports of RP with bilateral IDON in Chinese people. This case presents a six-year-old Chinese boy with RP who had an episode of IDON and was detected with a mutation in oral-facial-digital syndrome 1 (*OFD1*) gene.

## 2. Case Report

A six-year-old boy had quickly progressive binocular vision loss in 38 days. On June 26, 2015, he complained of being unable to see grids in a notebook. Nine days later, his visual acuity (VA) was too weak for him to walk by himself. His first-visited hospital could not give a clear diagnosis and his visual function continued to deteriorate. There were no complaints of pain in his eyes or with eye movement; there was no history of respiratory tract infection or recent vaccination. However, mild night blindness had been noticed one year before. His family denied a history of hereditary disease. His parents did not have a consanguineous marriage.

**Figure 1:**
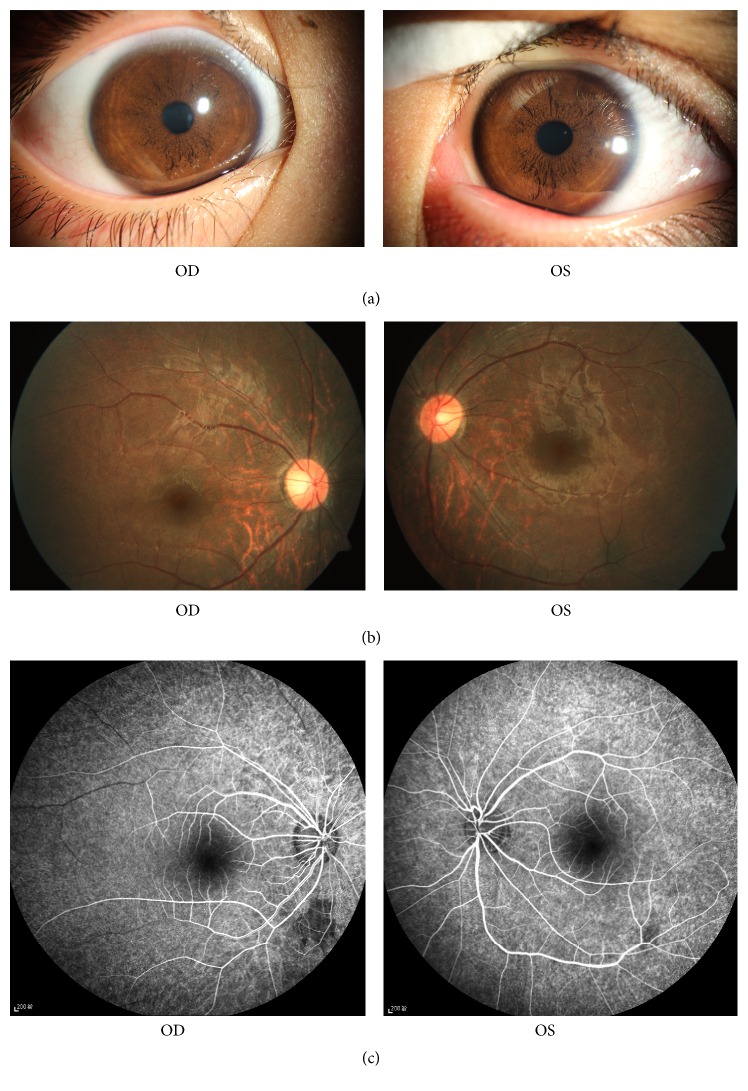
(a) shows the small black nodules on the surface of the iris in both eyes; (b) shows the pale optic disc, the mottling, and no bone spicule-like pigmentation fundus in both eyes; (c) shows the mottled lesions with hyper- and hypofluorescence in the posterior fundus and no leakage from the optic discs in both eyes.

On admission to our ophthalmic center on August 4, his VA was no light perception (NLP) bilaterally. His intraocular pressure was OD: 14.7 mmHg and OS: 12.0 mmHg. His pupillary diameters were 6 mm, and direct and indirect reflex disappeared in both eyes. There were many small black nodules on the surface of his bilaterally irises (Figure 1(a)). Both of his optic discs were pale and the disc edge was slightly blurred. The retinal vessels were attenuate. The midperipheral fundus appeared mottled bilaterally, while no bone spicule-like pigmentation was found (Figure 1(b)). Other results of physical examinations were normal.

**Figure 2:**
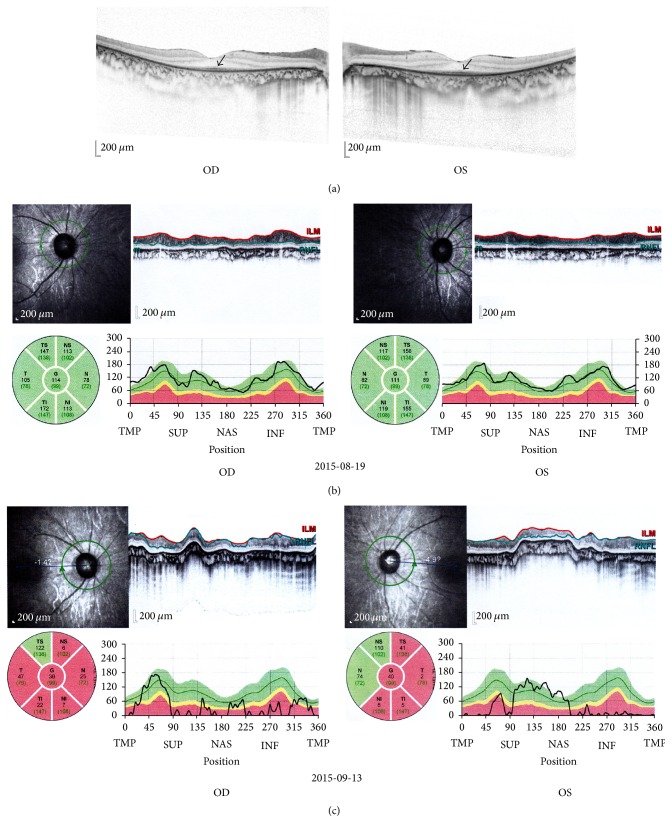
(a) shows thinning and atrophy of the outer retina and foveal sparing of the continuous ellipsoid zone of both eyes (arrows). (b) shows the RNFL thickness of both eyes was with of normal range on Aug. 19, 2015. (c) shows the RNFL thickness of both eyes became thinner in all quadrants on Sep. 13, 2015.

**Figure 3:**
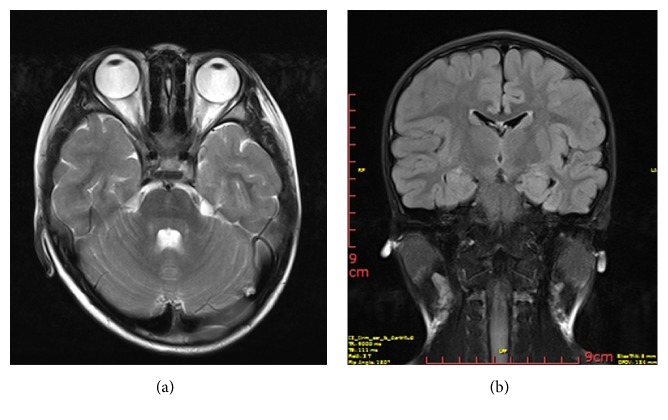
(a) shows the enlargement of bilateral optic nerves. (b) shows the demyelination spots of the right frontal and parietal lobes in brain.

Fluorescein fundus angiography showed mottled lesions with hyper- and hypofluorescence in the posterior fundus (Figure 1(c)). Optical coherence tomography (OCT) showed thinning and atrophy of the outer retinal segment, leaving the ellipsoid zone of the fovea intact (Figure 2(a)). The retinal nerve fiber layer (RNFL) of both eyes was within normal range (Figure 2(b)). The scotopic and photopic electroretinogram (ERG) response extinguished in both eyes. No flash visual-evoked potential (FVEP) response was detected. The magnetic resonance imaging (MRI) of his optic nerve showed bilateral optic nerve enlarged (Figure 3(a)) and that of brain showed several demyelination spots of the right frontal and parietal lobes (Figure 3(b)).

Anticardiolipin antibody and serous thyroglobulin antibodies were tested, 35.7 RU/mL and 129 U/mL, respectively. Aquaporin-4 antibody, the specific biomarker of neuromyelitis optica, was seronegative. Treponema pallidum hemagglutination test was seronegative and postprandial 2-hour blood glucose was 6.78 mmol/L, both within the normal range. The boy was well nourished and denied the history of beriberi, angular cheilitis, and anemia. Other immunologic tests (anti-double strand DNA, antinuclear antibodies, antineutrophil cytoplasmic antibodies, proliferating cell nuclear antigen, and human leukocyte antigen B27) were all negative.

**Figure 4:**
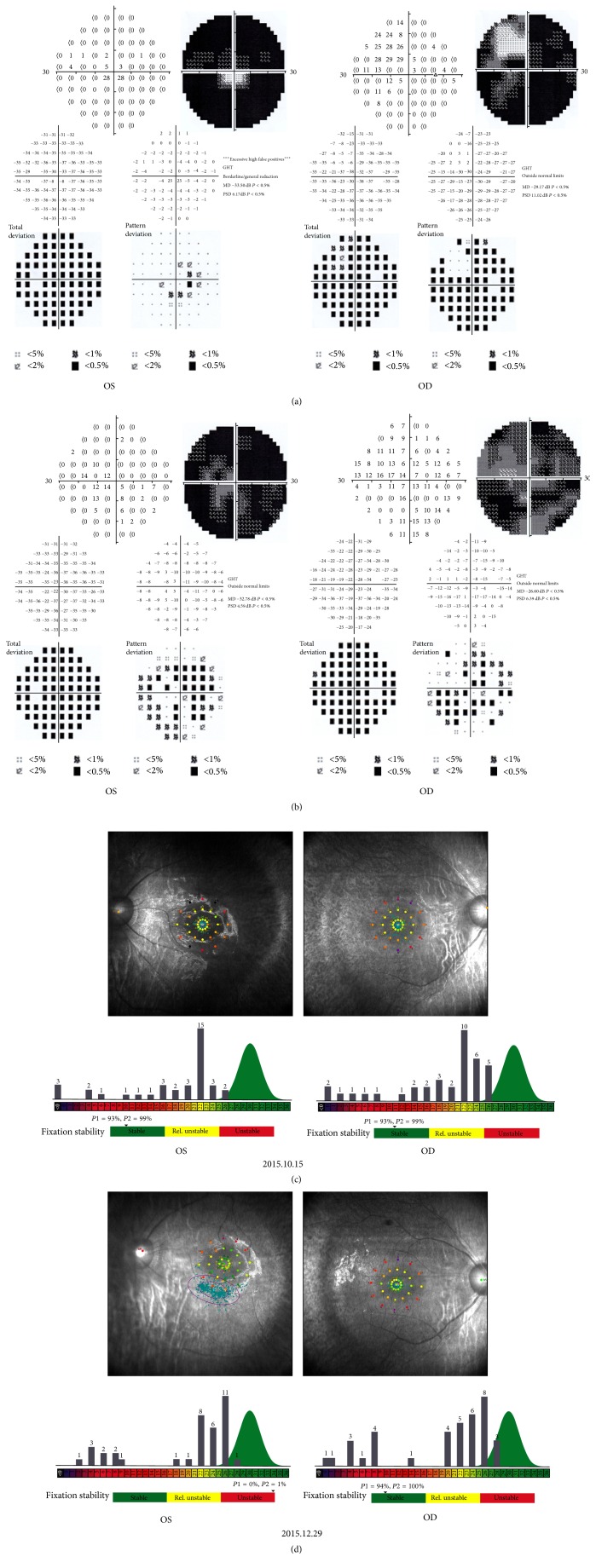
The Goldmann visual field of both eyes (a) shows the defect in all regions except the upper nasal quadrant of the right eye, on Oct. 15; (b) shows a diffuse improvement of the light sensitivity in the central visual field, on Dec. 29. The macular microvisual field of both eyes (c) shows the greatly damaged central visual field on Oct. 15; (d) shows an improved central visual field on Dec. 29.

The boy's condition was diagnosed as RP with IDON; then, he received corticosteroid therapy (oral administration of methylprednisolone by 20 mg/day for 14 days and then tapered off). After treatment, his best-corrected VA gradually increased to OD: 0.5 and OS: 0.4. Low vision Farnsworth PV-16 test revealed tritanopia in his both eyes. The OCT showed that the RNFL was thinner in all quadrants than in August (Figure 2(c)). Visual field examination showed diffuse constriction of peripheral visual field and a greatly damaged central visual field (Figures 4(a) and 4(c)).

Three months later, the central visual field showed diffuse improvement of light sensitivity (Figures 4(b) and 4(d)). VEP was normal in both eyes. ERG remained extinguished. The concentration of thyroglobulin antibodies and anticardiolipin antibody decreased to 66.1 U/mL to 12.5 RU/mL, respectively.

**Figure 5:**
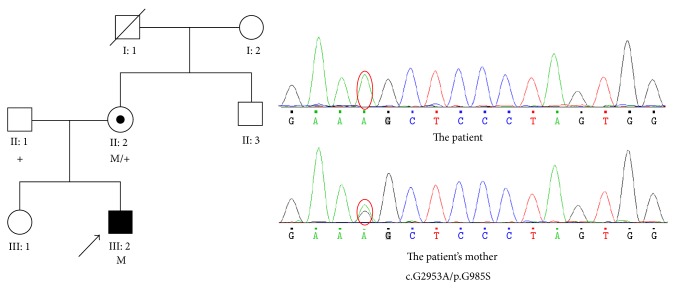
The sequence map, pedigree, and cosegregation analysis of the family with novel mutation of* OFD1*.

Targeted next-generation sequencing revealed a homozygous p.G985S mutation (c.G2953A) in* OFD1* gene, with X-linked recessive inheritance. Sanger sequencing confirmed the variant and it is absent in exome variant server and 1000 Genomes database. Parental testing confirmed that the mutation was from the boy's mother, a carrier who had no ocular phenotypes. The parents, grandparents, and mother's siblings of the patient all denied any kind of visual problem or night blindness. The pedigrees, cosegregation analyses, and sequence maps of the family are shown in Figure 5. This variant has not been reported associated with RP before.

## 3. Discussion

Rarely has RP with bilateral IDON been reported in the past. The only case report in literature was a 14-year-old Japanese girl who had RP, retrobulbar optic neuritis, and rhegmatogenous retinal detachment in the right eye simultaneously, without a candidate gene reported [2].

In our case, night blindness, mottled retina without bone spicule-like pigmentation, and extinguished scotopic ERG response in both eyes indicated RP without pigmentation. Progressive decrease in bilateral visual acuity, pale optic discs, abnormal VEP, loss of central visual field, and demyelinating spot in the brain MRI suggested IDON by excluding other optic neuropathies. The rise of the thyroglobulin antibodies and anticardiolipin antibody indicated abnormal autoimmunity. The rapid increase of visual function after corticosteroid treatment supports the diagnosis of IDON. Though the patient's condition improved after treatment, his RNFL was thinner in all quadrants. During the long-term course of RP, the RNFL of patients can be thinner [3]. While, in this patient, the edema caused by optic neuritis made the thickness within normal range, after anti-inflammatory therapy, edema subsided and the attenuation of RNFL manifested. As reported by Oishi et al., the decrease rate of RNFL was not associated with visual functions [3]. Central visual acuity have a close relationship with presence of the ellipsoid zone instead of the thickness of RNFL [4].

Leber hereditary optic neuropathy (LHON) is a mitochondrial disease that mostly causes blindness in young adult males. Continuous vision loss in LHON is far more common than simultaneous binocular vision loss, which was less than 10% [5]. In patients with LHON, no light perception is extremely rare. Pupillary light reflex of patients was relatively preserved compared with the extent of visual loss [6]. Moreover, corticosteroid therapy had no beneficial effect on LHON. In this case, although there is no available mitochondrial genetic test for the refusal of the patient's parents, the diagnosis of LHON was ruled out.

Retinitis pigmentosa can be inherited in autosomal dominant, autosomal recessive, and X-linked patterns [7–9]. In previous research, IDON was reportedly associated with some gene polymorphisms in Asian people [10]. The targeted sequencing revealed a homozygous p.G985S mutation in* OFD1* gene with X-linked patterns. Mutations in* OFD1* may lead to reduced levels of normal ciliopathy protein and be related to retinal degeneration. A deep intronic mutation in* OFD1* responsible for a severe form of X-linked RP was reported before [11]. The responsibility of the variant for the reduced expression of* OFD1* and the episode of optic neuritis need to be further confirmed.

It is hard to judge the reason for the visual acuity decrease in patients with RP and other complications. VA progressively decreased to NLP might happen in the later stage of severe RP. The presence of the ellipsoid zone is essential for preserved visual function in patients with RP [4]. The intact ellipsoid zone of the patient's fovea indicates other causes for the VA decrease. The suspicion of optic neuritis was raised by the rapid progression of the VA decrease, the demyelinating foci on the MRI, and the abnormality of the autoantibodies [12]. The rapid response to corticosteroid therapy and the recovery of VEP further confirmed the diagnosis.

RP with bilateral IDON had not been reported before in Chinese people. In this case, the diagnosis was delayed for nearly two months. The patient's optic nerves atrophied severely before he received correct treatment. Timely diagnosis of optic neuritis in patients with RP and prompt treatment are important to save visual function. The gene survey is useful to clarify the diagnosis and help understand its pathogenicity.
